# GASdb: a large-scale and comparative exploration database of glycosyl hydrolysis systems

**DOI:** 10.1186/1471-2180-10-69

**Published:** 2010-03-04

**Authors:** Fengfeng Zhou, Huiling Chen, Ying Xu

**Affiliations:** 1 Computational Systems Biology Laboratory, Department of Biochemistry and Molecular Biology, and Institute of Bioinformatics, University of Georgia, Athens, GA 30602, USA; 2 BioEnergy Science Center, Oak Ridge, TN, 37831, USA

## Abstract

**Background:**

The genomes of numerous cellulolytic organisms have been recently sequenced or in the pipeline of being sequenced. Analyses of these genomes as well as the recently sequenced metagenomes in a systematic manner could possibly lead to discoveries of novel biomass-degradation systems in nature.

**Description:**

We have identified 4,679 and 49,099 free acting glycosyl hydrolases with or without carbohydrate binding domains, respectively, by scanning through all the proteins in the UniProt Knowledgebase and the JGI Metagenome database. Cellulosome components were observed only in bacterial genomes, and 166 cellulosome-dependent glycosyl hydrolases were identified. We observed, from our analysis data, unexpected wide distributions of two less well-studied bacterial glycosyl hydrolysis systems in which glycosyl hydrolases may bind to the cell surface directly rather than through linking to surface anchoring proteins, or cellulosome complexes may bind to the cell surface by novel mechanisms other than the other used SLH domains. In addition, we found that animal-gut metagenomes are substantially enriched with novel glycosyl hydrolases.

**Conclusions:**

The identified biomass degradation systems through our large-scale search are organized into an easy-to-use database GASdb at http://csbl.bmb.uga.edu/~ffzhou/GASdb/, which should be useful to both experimental and computational biofuel researchers.

## Background

As a promising alternative energy source to fossil fuels, biofuels can be produced through degradation and fermentation of lignocellulosic biomass of plant cell walls [1,2]. A key challenge in converting biomass to fuels lies in the special structures of cell walls that plants have formed during evolution to resist decomposition from microbes and enzymes. It is this defense system of plants that makes their conversion to fuel difficult, which is known as the *biomass recalcitrance *problem [3]. Considerable efforts have been invested into searches for microbes, specifically cellulolytic microbes, which can effectively break down this defense system in plants.

Cellulolytic microbes degrade biomass through secreting glycosyl hydrolases, binding to the biomass using their carbohydrate binding domains (CBMs), and then cutting various chemical bonds of the biomass using their catalytic domains [4]. It has been observed that the catalytic efficiency of a glycosyl hydrolase (WGH) decreases when it does not have a CBM domain [5,6], compared to the ones with such a domain. While some microbes use directly multiple glycosyl hydrolases, independent of each other, for biomass degradation, other microbes use them in an organized fashion, i.e., orchestrating them into large protein complexes, called *cellulosomes*, through scaffolding (Sca) proteins. The former are called free acting hydrolases (FAC), and the latter called cellulosome dependent hydrolases (CDC) [4,7]. Some anaerobic microbes use both systems for biomass degradation [7] while most of the other cellulolytic microbes use only one of them. When degrading biomasses, cellulosomes are generally attached to their host cell surfaces by binding to the cell surface anchoring (SLH) proteins [8]. The general observation has been that cellulosomes are more efficient in degradation of biomass into short-chain sugars than free acting cellulases [8]. Our goal in this computational study is to identify and characterize all the component proteins of the biomass degradation system in an organism, which is called the *glydrome *of the organism.

We have systematically re-annotated and analyzed the functional domains and signal peptides of all the proteins in the UniProt Knowledgebase and the JGI Metagenome database, aiming to identify novel glycosyl hydrolases or novel mechanisms for biomass degradation. Based on their domain compositions, we have classified all the identified glydrome components into five categories, namely FAC, WGH, CDC, SLH and Sca. To our surprise, two less well-studied glycosyl hydrolysis systems were found to be widely distributed in 63 bacterial genomes, in which (a) glycosyl hydrolases may bind directly to the cell surfaces by their own cell surface anchoring domains rather than through those in the cell surface anchoring proteins or (b) cellulosome complexes may bind to the cell surface through novel mechanisms other than the SLH domains, respectively, as previously observed. Our analyses also suggest that animal-gut metagenomes are significantly enriched with novel glycosyl hydrolases. All the identified glydrome elements are organized into an easy-to-use database, GASdb, at http://csbl.bmb.uga.edu/~ffzhou/GASdb/.

## Construction and content

### Data sources

We downloaded the UniProt Knowledgebase release 14.8 (Feb 10, 2009) [9] with 7,754,276 proteins, and all the 46 metagenomes from the JGI IMG/M database [10] with 1,504,133 proteins. The three simulated metagenomes in the database were excluded from our analysis.

The operon annotations were downloaded from DOOR [11,12].

### Annotation and database construction

We have identified the signal peptides and analyzed the functional domains for all the proteins using SignalP version 3.0 [13,14] and Pfam version 23.0 [15]. A protein is defined as a cell surface anchoring protein, if it has one SLH domain and one Cohesin domain; a scaffolding has at least three Cohesin domains or one Cohesin domain and one carbohydrate binding domain; a cellulosome dependent catalytic protein has one catalytic domain and one dockerin domain; a free acting catalytic protein has one catalytic domain and one CBM domain; and all the other proteins with one catalytic domain are defined as weak catalytic proteins.

We calculated the percentages of glydrome components in genomes with at least 1,000 proteins only, since most of the others may not have completely sequenced. Three dimension protein structures were predicted using LOMETS [16]. The protein's Gene Ontology annotations were predicted using PFP [17].

To make the annotated glydromes easy to be accessed, a database GASdb was constructed using PHP scripting language.

### Identified glydromes in bacteria

4,616 FACs are identified from the 7.75 million proteins in the UniProt Knowledgebase (release 14.8) [see Additional file 1]. The majority of them, 2,774 (61.71%), are from bacterial genomes. 1,019 FACs are found in the phylum *Firmicutes*, of which are a number of well-studied cellulolytic organisms such as *Anaerocellum thermophilum *[18], *Caldicellulosiruptor saccharolyticus *[19] and *Clostridium thermocellum *[20,21]. In addition, a large number of FACs are found in each of the two other phyla, namely *Bacteroidetes *(342 FACs) and *Actinobacteria *(425 FACs). Overall, these three phyla harbour 64.38% (~1,786/2,774) of our identified bacterial FACs, comparing to 25.12% of all the bacterial genomes covered by these phyla.

The previous observation has been that a functional cellulosome consists of at least one cell surface anchoring protein with SLH domains, at least one scaffolding protein and a number of cellulosome dependent glycosyl hydrolases [3,8,22,23]. Our search and analysis results indicate that novel biomass-degradation mechanisms may exist in the genomes or metagenomes that we analyzed, the details of which will need further studies. For example, *Clostridium acetobutylicum *was known to encode a scaffolding protein and a few cellulosome dependent enzymes, but it is not clear how the cellulosome is anchored to the cell surface [24,25] as no SLH domains were identified in the genome [see Additional file 1]. The similar question holds for the other four *Firmicutes*, i.e. *Clostridium cellulolyticum*, *Clostridium cellulovorans*, *Clostridium josui *and *Ruminococcus flavefaciens*. We did not expect that the scaffolding proteins in all these genomes except for *Ruminococcus flavefaciens *encode a domain of unknown function (PF03442: DUF291). Our data supports the previous observation that the four DUF291 domains in the *C. cellulovorans *scaffolding *CbpA *are possibly involved in anchoring the cellulosome on the cell surface [26].

**Figure 1:**
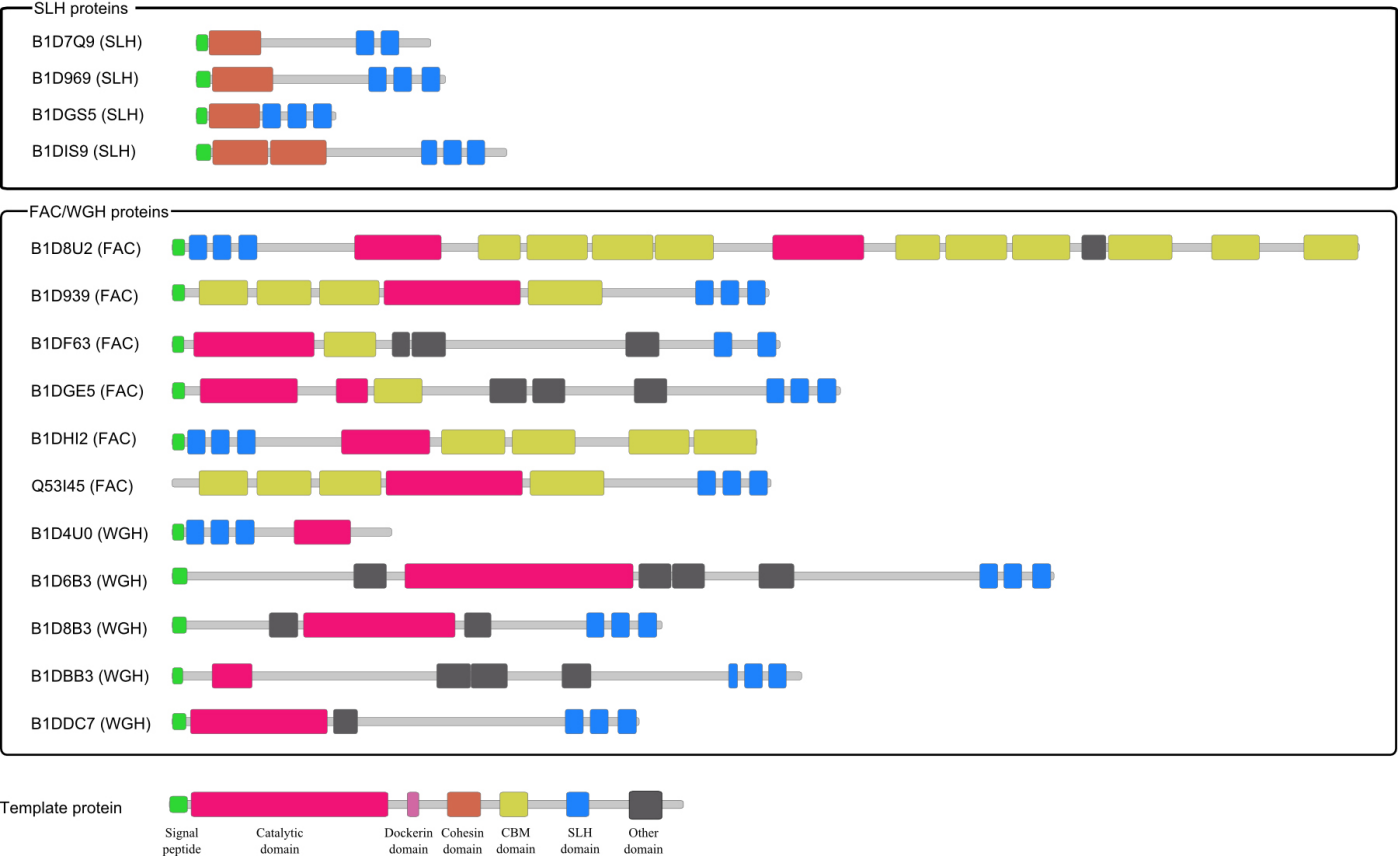
**Domain structures of four SLH proteins and eleven glycosyl hydrolases with SLH domains in *Paenibacillus *sp. JDR-2**.

A somewhat unusual glydrome was identified in *Paenibacillus *sp. JDR-2 of phylum *Firmicutes*. *Paenibacillus *sp. JDR-2 was known to encode modular xylanases [27,28] as shown in Figure 1. It is surprising to find 4 SLH proteins, i.e. B1D7Q9, B1D969, B1DGS5 and B1DIS9, but no other cellulosome components in *Paenibacillus *sp. JDR-2. Our search did not find any dockerin domains in the genome, suggesting the possibility that the organism uses an unknown biomass-degradation mechanism. In addition our search also identified SLH domains in 6 FACs and 5 WGHs of this organism, as shown in Figure 1. The superfamily of Ig-like fold domains are found in varieties of cell surface proteins [29], and the existence of them (Big_2, Big_4, and fn3, etc) in the aforementioned proteins further supports that they may anchor to the cell surface.

Overall a large number of glycosyl hydrolases without carbohydrate binding domains or dockerin domains were identified in the bacterial genomes. More than 2,000 WGHs are found in each of the following four phyla, *Proteobacteria *(10,442 WGHs), *Firmicutes *(6,084 WGHs), *Bacteroidetes *(2,885 WGHs) and *Actinobacteria *(2,371 WGHs). Top 3 bacterial genomes with the highest percentages of glycosyl hydrolases (FACs, WGHs and CDCs) are *Bacteroides intestinalis *DSM 17393 (5.11%), *Bacteroides ovatus *ATCC 8483 (4.49%) and *Bacteroides thetaiotaomicron *(4.40%).

### Identified glydromes in archaea

18 FACs are identified in six genera of *Archaea*, i.e. *Thermococcus*, *Halobacterium*, *Pyrococcus*, *Thermofilum*, *Caldivirga *and *Haloferax *[see Additional file 1], covering 11 genomes. Each of these 11 archaeal genomes encodes 1-3 FACs together with up to 28 WGHs. FACs were known to be encoded in four archaeal genomes, i.e. *Halobacterium mediterranei *[30], *Pyrococcus furiosus *[31,32], *Pyrococcus kodakaraensis *[33] and *Ferroplasma acidiphilum strain Y *[34]. Three of them are in our list. The glycosyl hydrolase in *Ferroplasma acidiphilum strain Y *was missed in our database since our annotation is based on the knowledge from the two databases, CAZy [35] and Pfam [15], neither of which includes this enzyme. 14 of the 18 identified FACs are homologous to each other with NCBI BLAST *E-values *< 1e-132 in different species of the same genus, suggesting that these enzymes have been in the 11 archaeal genomes at least before the divergence of these species.

385 proteins are annotated as WGHs in the 93 genomes from 30 archaeal genera. No cellulosome components were found in any of the archaeal genomes.

### Identified glydromes in eukaryota

1,824 FACs are found in the 1,668 eukaryotic genomes covering 23 phyla, 62.23% (1,135/1,824) of which were from fungal genomes. A green plant phylum *Streptophyta *(664 FACs) contributes to 36.40% of the FACs. All the other phyla encode less than 100 FACs. Four plant genomes encode more than 45 FACs, and they are *Oryza sativa sp japonica *(*Rice*) (99 FACs), *Vitis vinifera *(*Grape*) (71 FACs), *Arabidopsis thaliana *(*Mouse-ear cress*) (65 FACs) and *Zea mays *(*Maize*) (47 FACs). The other 25 non-fungi FACs are encoded in 5 unicellular algae and 6 animal genomes.

17,048 WGHs are found in the 1,668 eukaryotic genomes. The top three phyla in the numbers of FACs are also top three in the numbers of WGHs; and 2,328, 5,444 and 5,171 WGHs are encoded in three phyla *Arthropoda*, *Ascomycota *and *Streptophyta*, respectively. The top four eukaryotic genomes in the numbers of WGHs are from the phylum *Streptophyta*, and they are *Oryza sativa sp japonica *(*Rice*) (828 WGHs), *Arabidopsis thaliana *(*Mouse-ear cress*) (678 WGHs), *Vitis vinifera *(*Grape*) (602 WGHs) and *Zea mays *(*Maize*) (284 WGHs).

It is interesting to observe that there are 272 and 224 WGHs in the human and mouse genomes, respectively. Besides two other plant genomes, i.e. *Oryza sativa subsp. indica *(*Rice*) (258 WGHs) and *Physcomitrella patens sp patens *(*Moss*) (226 WGHs), all the other 6 eukaryotic genomes encoding more than 200 WGHs are from the fungal phylum *Ascomycota*. No cellulosome components were identified in the eukaryotic genomes. 200 (~73.53%) human WGHs are homologous to mouse WGHs with NCBI BLAST *E-values *< e-23. So the majority of these enzymes have been in the genomes of human and mouse at least before their divergence 75 million years ago [36].

### Identified glydromes in metagenomes

Overall, 63 FACs and 6,072 WGHs are found in 42 metagenomes except for TM7b which was sampled from the human mouth. The top two metagenomes in the numbers of glycosyl hydrolases are from termite guts (12 FACs and 1,150 WGHs) and diversa silage soil (13 FACs and 820 WGHs). Since the number of proteins in metagenomes varies from 452 in termite gut fosmids to 185,274 in the diversa silage soil, we calculated the percentage of the glycosyl hydrolases in each metagenome. On average, 0.65% of a metagenome encode glycosyl hydrolases. We noted that all the metagenomes with more than 1% encoding glycosyl hydrolases are from the animal guts (including human, mouse and termite). This is confirmed by an independent study using BLAST mapping [37]. No cellulosome components were identified in any metagenome.

## Utility

### The query interface of GASdb

**Figure 2:**
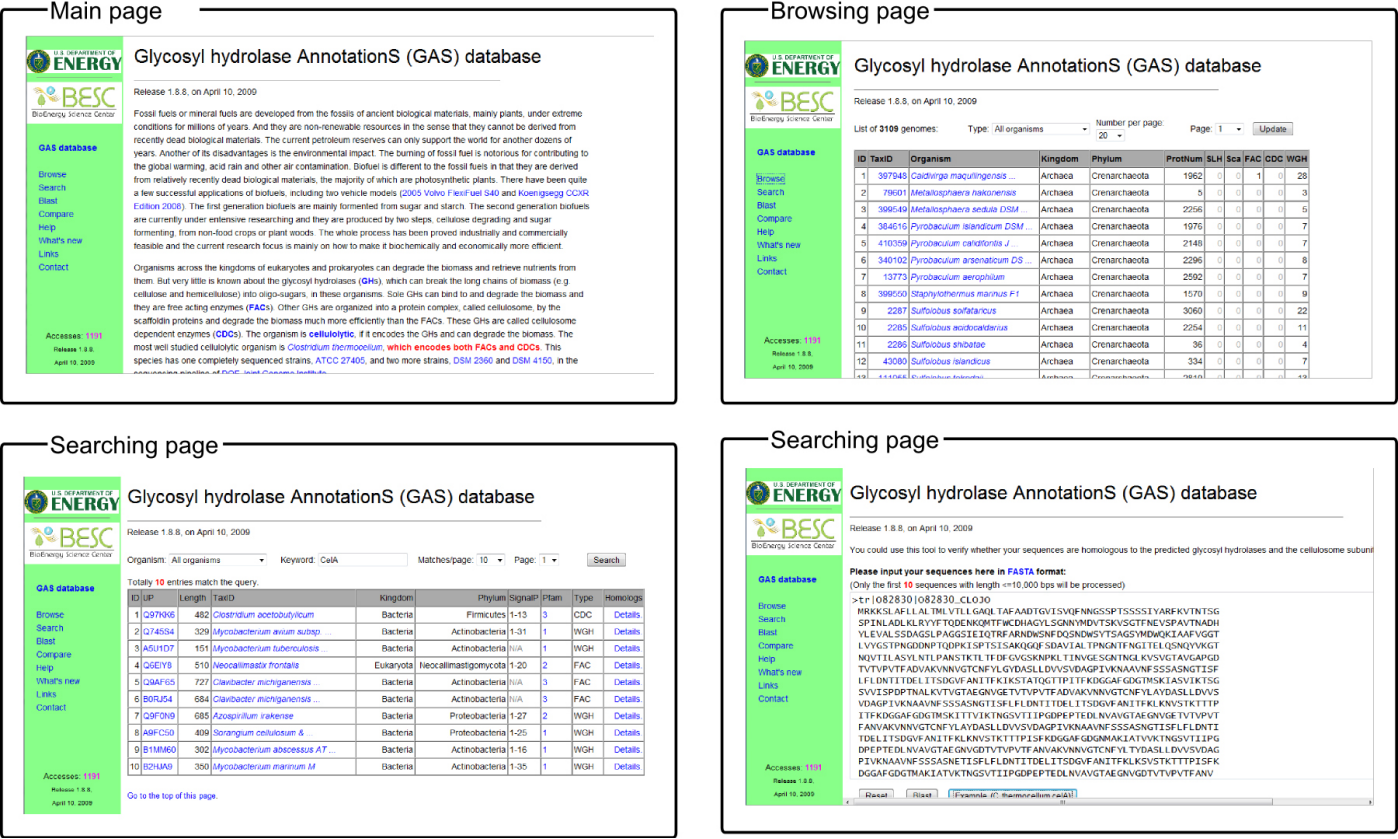
**The database interfaces: the main page, the browsing page, the searching page, and the BLAST page**.

**Figure 3:**
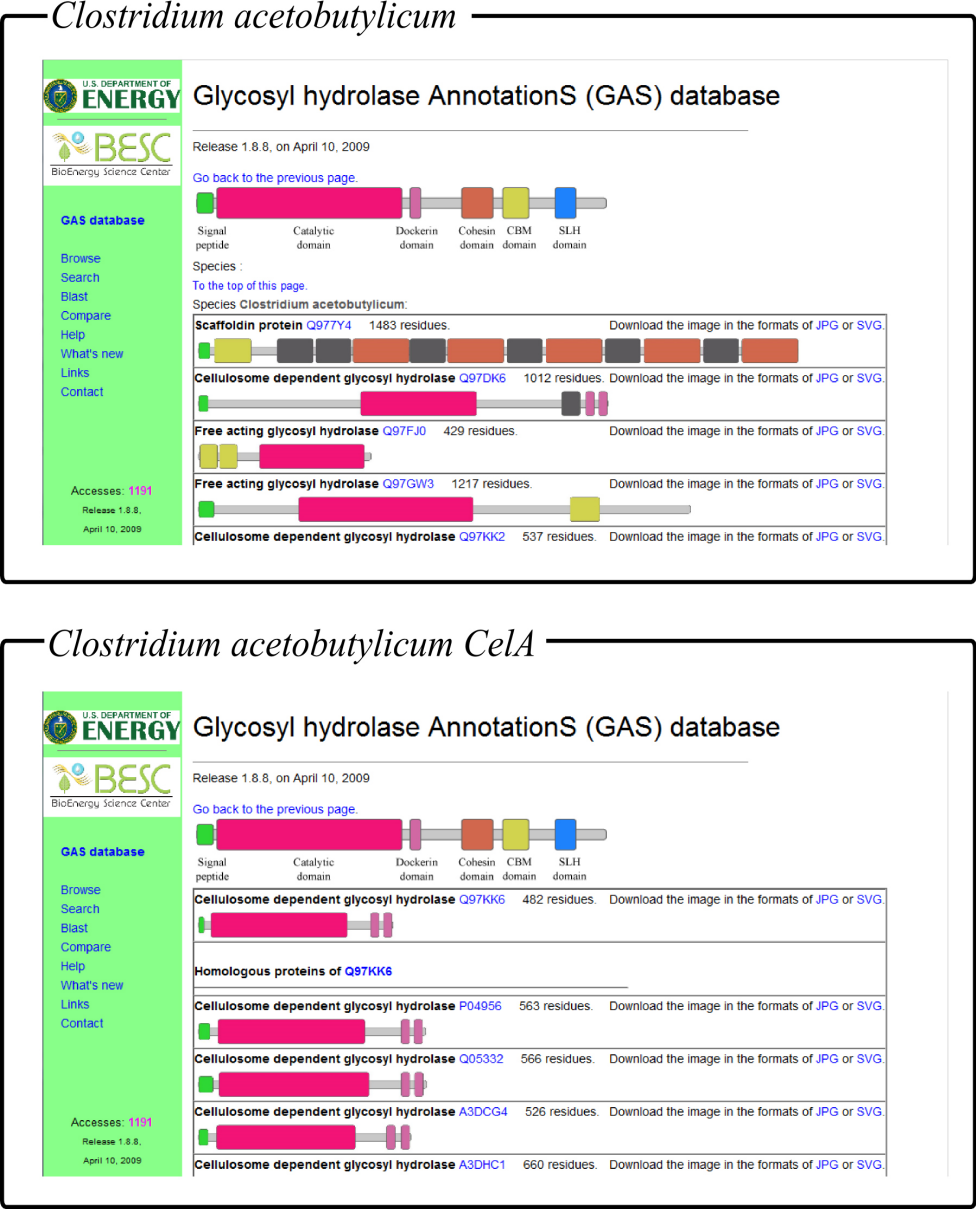
**The displaying pages for the domain architectures of the glydrome of *Clostridium acetobutylicum*, and domain architectures of the protein *Clostridium acetobutylicum *CelA and its homolog**.

All the annotated glydromes were organized into an easy-to-use database GASdb (Figure 2). A user can find the proteins of interest through browsing, and searching using keywords or BLAST. The overall organization of each glydrome can be displayed; and the high resolution images of each protein can be downloaded for the publication purpose, as shown in Figure 3. A user can also display the signal peptide and functional domains of a given protein and its homologs using BLAST with E-value cutoff 1e-20, as shown in Figure 3.

### The comparative analysis interface of GASdb

**Figure 4:**
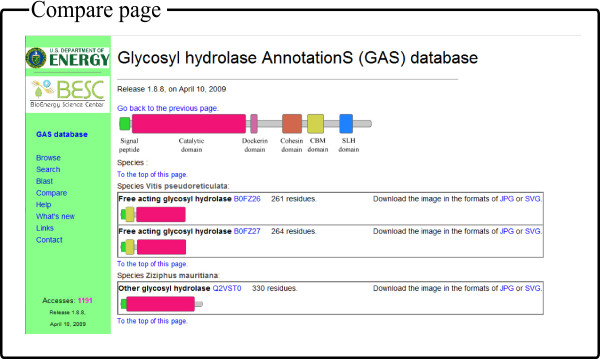
**The comparative analyzing interface of GASdb with *Vitis pseudoreticulata *and *Ziziphus mauritiana *as an example**.

The glydromes of multiple genomes can be illustrated in the Compare interface. First, the user needs to find the genome(s) of interest using keywords through the Compare interface. Then one or multiple genomes can be selected from the left panel in Figure 4, and added to the right panel for final display. The user can also remove some genomes from the right panel. The signal peptides and functional domains of proteins in the selected glydromes in the right panel will be displayed in the next page by clicking the Compare button, as shown in Figure 4.

## Discussion

The majority (52.90%) of glycosyl hydrolases (including FACs, CDCs and WGHs) in our database are encoded by the 1,771 bacterial genomes. The 1,668 eukaryotic genomes contribute 34.98% of the total glycosyl hydrolases. So the glycosyl hydrolases are much more enriched in bacteria than in eukaryotes, considering the substantially larger sizes of eukaryotic genomes. Cellulosome components are observed only in *Firmicutes*, except for the CDC *xynB *(Q7UF11) from *Rhodopirellula baltica*. All the other glycosyl hydrolases do not have dockerin domains, and were annotated as FACs or WGHs. Although the catalytic domain and the CBM domain of a glycosyl hydrolase can function independently, the CBM domain is known to play an important role in the catalytic efficiency of glycosyl hydrolase [5,6]. So the annotated FACs may have higher catalytic efficiency.

A cell surface anchoring protein binds to the cell surface through its two or three SLH domains, and binds to the cellulosome scaffolding proteins together with the CDCs through the interacting pairs of cohesin domains and dockerin domains. It is unexpected to find SLH domains in additional 5 FACs and 5 WGHs of *Paenibacillus *sp. JDR-2, as the only previous observation related to this is Q53I45 (*XynA*) in *Paenibacillus *sp. JDR-2 genome [28]. We believe that these glycosyl hydrolases may bind to the cell surface through their own SLH domains, as *Paenibacillus *sp. JDR-2 encodes SLH proteins but no scaffoldings or CDCs. It would be interesting to study how *Paenibacillus *sp. JDR-2 acquired the SLH proteins or lost the other cellulosome components. We noticed that this is not a unique feature of *Paenibacillus *sp. JDR-2, as there are 26 FACs and 52 WGHs with SLH domains in the other organisms, all of which are bacteria, except for the moss *Physcomitrella patens*. Many of these enzymes have been experimentally confirmed to anchor on the cell surfaces through the SLH domains, e.g. the cell surface xylanase *xyn5 *(Q8GHJ4) from *Paenibacillus *sp. W-61 [38,39], the extra-cellular endoglucanase *celA *(Q9ZA17) from *Thermoanaerobacterium polysaccharolyticum *[40] and the endoxylanase (Q60043) from *Thermoanaerobacterium *sp. strain JW/SL-YS 485 [41].

**Figure 5:**
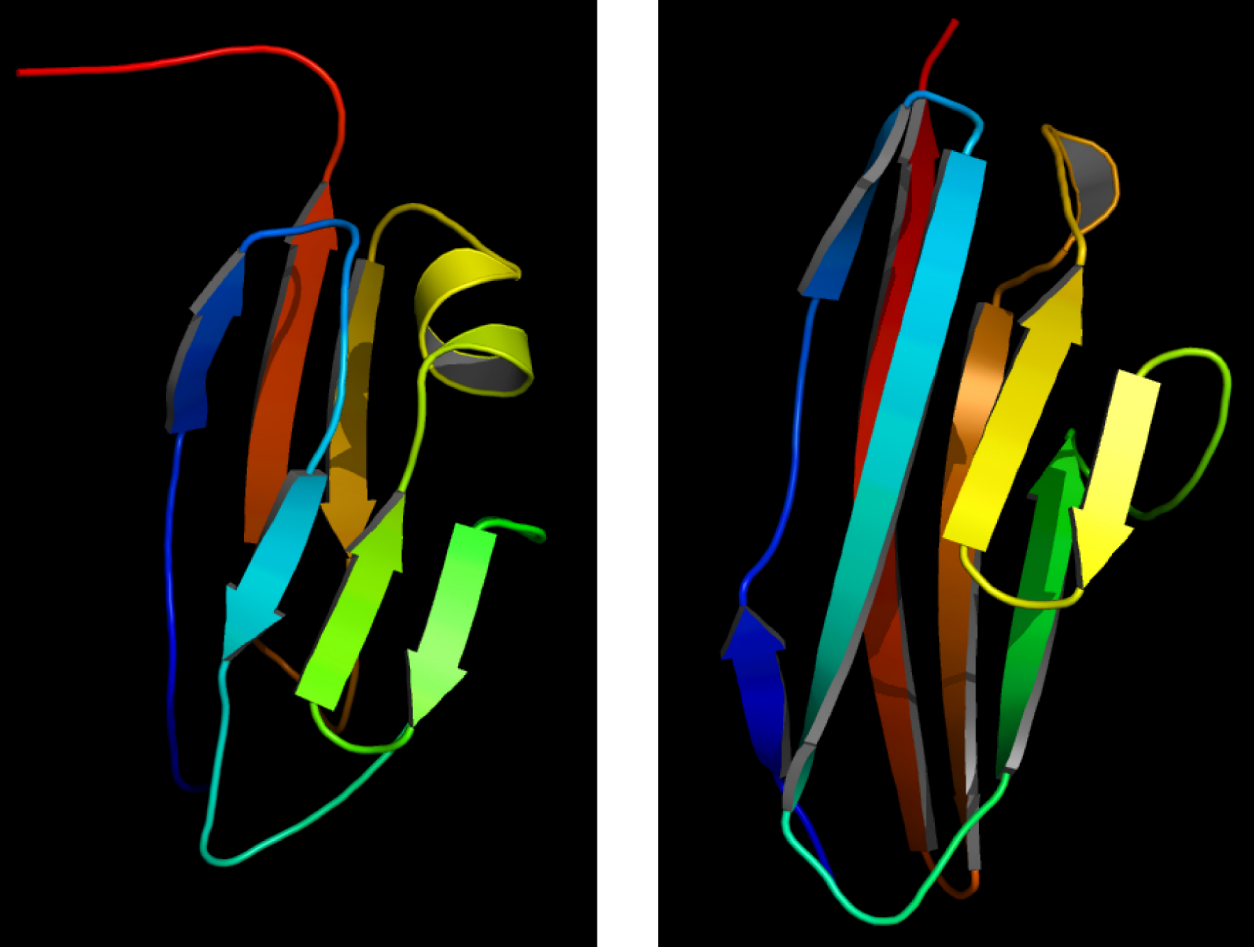
**Top two predicted structures of the first DUF291 (PF03442) domain of the scaffolding Q977Y4 of the *Clostridium acetobutylicum *glydrome, with templates 1ehxa and 1cs6a, respectively**.

Cellulosomes could be linked to the cell surfaces using novel mechanisms other than through the typically used SLH domains as our data indicate. Five *Firmicutes *encode scaffolding proteins and CDCs but no recognizable SLH domains, a key feature for the cell surface anchoring proteins. The cellulosomes were observed to anchor on the cell surfaces in *Clostridium cellulolyticum *[22], *Clostridium cellulovorans *[42] and *Ruminococcus flavefaciens *[7]. But the detailed mechanisms remain to be known. The cellulosomes in *Clostridium acetobutylicum *and *Clostridium josui *may also be linked to the cell surfaces through some unknown mechanisms. Our analysis suggests that the domain of unknown function DUF291 (PF03442) might be involved in attaching these cellulosomes to the cell surfaces. We predicted the 3D structure of the first DUF291 domain in the scaffolding Q977Y4 of the *Clostridium acetobutylicum *glydrome, as shown in Figure 5. The first template (1EHX) does not show functional implication, while the second one (1CS6) is involved in cell adhesion [43,44]. The difference between the two predicted structures of the DUF291 domain is similar to each other with RMSD~2.7 A and TM score 0.6 using TM-align [45,46].

We collected 41 proteins encoded in the same operons with the components of *Clostridium acetobutylicum *glydrome but not in our GASdb. 16 of these proteins cover the following functional categories: binding (GO:0005488), catalytic activity (GO:0003824) and transporter activity (GO:0005215), and the remaining 25 are hypothetical or uncharacterized proteins. Only five proteins were annotated to be involved in the glycosyl hydrolysis, e.g. carbohydrate binding (GO:0030246) or hydrolase activity (GO:0016787). Three of the five proteins missed in our GASdb, i.e. Q97EZ1, Q97FI9 and Q97TI3, do not have recognizable Pfam domains related to the glycosyl hydrolysis. Q97TP4 is annotated to be an esterase (family 4 CE). The cellulosome integrating protein Q97KK4 has only one Cohesin domain occupying ~77.35% (140/181) of its total length, and might have been inactivated by domain deletion.

In general, the glycosyl hydrolases and the cellulosome components attack the biomass after they are secreted outside the cells and properly assembled [23,47], and hence we would expect that they have certain signal peptides. However the majority of the annotated glycosyl hydrolases do not have any signal peptides, based on the predictions of SignalP 3.0 [13,14]. We found that over 65% of WGHs across all organisms except for Eukaryota do not have predicted signal peptides suggesting the possibility of these proteins using a novel secretion mechanism.

The ratio between the numbers of WGHs and FACs in a glydrome tends to be no more than 30. We calculated this ratio for each glydrome in a genome or metagenome with at least 1,000 proteins and at least one FAC and one WGH. We observed that the averaged ratios between the numbers of WGHs and FACs are 9.98, 12.55 and 14.40 for archaea, bacteria and eukaryota, with standard derivations 8.22, 16.65 and 12.25, respectively. Overall, over 90% of the glydromes in archaea, bacteria and eukaryota are lower than 30 in this ratio, respectively. It is surprising to find that the metagenomes encode 95.38 times more WGHs than FACs but no cellulosome components. We speculate that there may be some novel CBM domains being used by these WGHs in these metagenomes. An alternative hypothesis could be that microbes in a community generously secrete WGHs to degrade biomass and live on the hydrolysis products in the nearby regions only.

## Conclusions

We conducted the first large-scale annotation of glydromes in all the sequenced genomes and metagenomes. We have made a number of interesting observations about glydromes of the sequences genomes and metagenomes. Among them, two less well-studied glydromes were observed in dozens of organisms, which are A) glycosyl hydrolases were found to have cell surface anchoring domains and can bind to the cell surfaces by themselves; and B) *Clostridium acetobutylicum *and four other bacteria from the phylum *Firmicutes *encode all cellulosome components except for the cell surface anchoring proteins SLHs, suggesting that the cellulosomes may have link to the cell surfaces through some novel mechanisms. Individual cases have been experimentally observed, but further studies are needed to uncover the underlining mechanisms and how they evolved into the current glydrome structures. Our data also suggested that the animal gut metagenomes are rich in novel glycosyl hydrolases, providing new targets for further experimental studies.

## Availability and requirements

Project name: GASdb;

Project home page: http://csbl.bmb.uga.edu/~ffzhou/GASdb/;

Operating systems: Platform independent;

Programming language: Perl, PHP, Apache

License: none;

Restrictions to use by non-academics: none.

## Authors' contributions

YX wrote and polished the manuscript, and served as the principle investigator of the project. FZ performed the identification and annotation of the data, constructed the web site and wrote the manuscript. HC conducted the functional characterization based on structural information. All authors have read and approved the final submitted version of this manuscript.

## Supplementary Material

Additional file 1 **The numbers of annotated glydrome components in each organism**. A summary of the numbers of the annotated glydrome components in each organism. Click here for file

## References

[B1] Galperin MY The quest for biofuels fuels genome sequencing Environ Microbiol 2008 10 10 2471 2475 10.1111/j.1462-2920.2008.01754.x 18821974 PMC2613243

[B2] Rubin EM Genomics of cellulosic biofuels Nature 2008 454 7206 841 845 10.1038/nature07190 18704079

[B3] Himmel ME Biomass Recalcitrance: Deconstructing the Plant Cell Wall For Bioenergy 2008 Blackwell Publishing

[B4] Doi RH Cellulases of mesophilic microorganisms: cellulosome and noncellulosome producers Ann N Y Acad Sci 2008 1125 267 279 10.1196/annals.1419.002 18096849

[B5] Arai T Araki R Tanaka A Karita S Kimura T Sakka K Ohmiya K Characterization of a cellulase containing a family 30 carbohydrate-binding module (CBM) derived from Clostridium thermocellum CelJ: importance of the CBM to cellulose hydrolysis J Bacteriol 2003 185 2 504 512 10.1128/JB.185.2.504-512.2003 12511497 PMC145318

[B6] Arai T Ohara H Karita S Kimura T Sakka K Ohmiya K Sequence of celQ and properties of celQ, a component of the Clostridium thermocellum cellulosome Appl Microbiol Biotechnol 2001 57 5-6 660 666 10.1007/s00253-001-0832-4 11778875

[B7] Vanfossen AL Lewis DL Nichols JD Kelly RM Polysaccharide degradation and synthesis by extremely thermophilic anaerobes Ann N Y Acad Sci 2008 1125 322 337 10.1196/annals.1419.017 18378602

[B8] Bayer EA Lamed R White BA Flint HJ From cellulosomes to cellulosomics Chem Rec 2008 8 6 364 377 10.1002/tcr.20160 19107866

[B9] UniProt_Consortium The universal protein resource (UniProt) Nucleic Acids Res 2008 36 Database D190 195 18045787 10.1093/nar/gkm895 PMC2238893

[B10] Markowitz VM Ivanova NN Szeto E Palaniappan K Chu K Dalevi D Chen IM Grechkin Y Dubchak I Anderson I IMG/M: a data management and analysis system for metagenomes Nucleic Acids Res 2008 36 Database D534 538 17932063 10.1093/nar/gkm869 PMC2238950

[B11] Mao F Dam P Chou J Olman V Xu Y DOOR: a database for prokaryotic operons Nucleic Acids Res 2009 37 Database D459 463 10.1093/nar/gkn757 18988623 PMC2686520

[B12] Dam P Olman V Harris K Su Z Xu Y Operon prediction using both genome-specific and general genomic information Nucleic Acids Res 2007 35 1 288 298 10.1093/nar/gkl1018 17170009 PMC1802555

[B13] Emanuelsson O Brunak S von Heijne G Nielsen H Locating proteins in the cell using TargetP, SignalP and related tools Nat Protoc 2007 2 4 953 971 10.1038/nprot.2007.131 17446895

[B14] Bendtsen JD Nielsen H von Heijne G Brunak S Improved prediction of signal peptides: SignalP 3.0 J Mol Biol 2004 340 4 783 795 10.1016/j.jmb.2004.05.028 15223320

[B15] Finn RD Tate J Mistry J Coggill PC Sammut SJ Hotz HR Ceric G Forslund K Eddy SR Sonnhammer EL The Pfam protein families database Nucleic Acids Res 2008 36 Database D281 288 18039703 10.1093/nar/gkm960 PMC2238907

[B16] Wu S Zhang Y LOMETS: a local meta-threading-server for protein structure prediction Nucleic Acids Res 2007 35 10 3375 3382 10.1093/nar/gkm251 17478507 PMC1904280

[B17] Hawkins T Luban S Kihara D Enhanced automated function prediction using distantly related sequences and contextual association by PFP Protein Sci 2006 15 6 1550 1556 10.1110/ps.062153506 16672240 PMC2242549

[B18] Zverlov V Mahr S Riedel K Bronnenmeier K Properties and gene structure of a bifunctional cellulolytic enzyme (CelA) from the extreme thermophile 'Anaerocellum thermophilum' with separate glycosyl hydrolase family 9 and 48 catalytic domains Microbiology 1998 144 Pt 2 457 465 10.1099/00221287-144-2-457 9493383

[B19] Gibbs MD Reeves RA Farrington GK Anderson P Williams DP Bergquist PL Multidomain and multifunctional glycosyl hydrolases from the extreme thermophile Caldicellulosiruptor isolate Tok7B.1 Curr Microbiol 2000 40 5 333 340 10.1007/s002849910066 10706665

[B20] Berger E Zhang D Zverlov VV Schwarz WH Two noncellulosomal cellulases of Clostridium thermocellum, Cel9I and Cel48Y, hydrolyse crystalline cellulose synergistically FEMS Microbiol Lett 2007 268 2 194 201 10.1111/j.1574-6968.2006.00583.x 17227469

[B21] Fuchs KP Zverlov VV Velikodvorskaya GA Lottspeich F Schwarz WH Lic16A of Clostridium thermocellum, a non-cellulosomal, highly complex endo-beta-1,3-glucanase bound to the outer cell surface Microbiology 2003 149 Pt 4 1021 1031 10.1099/mic.0.26153-0 12686644

[B22] Belaich JP Tardif C Belaich A Gaudin C The cellulolytic system of Clostridium cellulolyticum J Biotechnol 1997 57 1-3 3 14 10.1016/S0168-1656(97)00085-0 9335163

[B23] Gilbert HJ Cellulosomes: microbial nanomachines that display plasticity in quaternary structure Mol Microbiol 2007 63 6 1568 1576 10.1111/j.1365-2958.2007.05640.x 17367380

[B24] Land PW Monaghan AP Abnormal development of zinc-containing cortical circuits in the absence of the transcription factor Tailless Brain Res Dev Brain Res 2005 158 1-2 97 101 10.1016/j.devbrainres.2005.04.006 15950290 PMC2724001

[B25] Sabathe F Belaich A Soucaille P Characterization of the cellulolytic complex (cellulosome) of Clostridium acetobutylicum FEMS Microbiol Lett 2002 217 1 15 22 10.1111/j.1574-6968.2002.tb11450.x 12445640

[B26] Taramu Y Liu C Ichi-Ichi A Malburg L Doi R Shimada K, Ohmiya K, Kobayashi Y, Hoshino S, Sakka K, Karita S The Clostridium cellulovorans cellulosome and non-cellulosomal cellulases Genetics Biochemistry and Ecology of Cellulose Degradation 1998 Tokyo: Uni Publishers Co 488 494

[B27] Chow V Nong G Preston JF Structure, function, and regulation of the aldouronate utilization gene cluster from Paenibacillus sp. strain JDR-2 J Bacteriol 2007 189 24 8863 8870 10.1128/JB.01141-07 17921311 PMC2168633

[B28] Stjohn FJ Rice JD Preston JF Paenibacillus sp. strain JDR-2 and XynA1: a novel system for methylglucuronoxylan utilization Appl Environ Microbiol 2006 72 2 1496 1506 10.1128/AEM.72.2.1496-1506.2006 16461704 PMC1392964

[B29] Kelly G Prasannan S Daniell S Fleming K Frankel G Dougan G Connerton I Matthews S Structure of the cell-adhesion fragment of intimin from enteropathogenic Escherichia coli Nat Struct Biol 1999 6 4 313 318 10.1038/7545 10201396

[B30] Holmes ML Dyall-Smith ML Sequence and expression of a halobacterial beta-galactosidase gene Mol Microbiol 2000 36 1 114 122 10.1046/j.1365-2958.2000.01832.x 10760168

[B31] Sybesma W Starrenburg M Kleerebezem M Mierau I de Vos WM Hugenholtz J Increased production of folate by metabolic engineering of Lactococcus lactis Appl Environ Microbiol 2003 69 6 3069 3076 10.1128/AEM.69.6.3069-3076.2003 12788700 PMC161528

[B32] Kaper T Lebbink JH Pouwels J Kopp J Schulz GE Oost J van der de Vos WM Comparative structural analysis and substrate specificity engineering of the hyperthermostable beta-glucosidase CelB from Pyrococcus furiosus Biochemistry 2000 39 17 4963 4970 10.1021/bi992463r 10819960

[B33] Tanaka T Fukui T Atomi H Imanaka T Characterization of an exo-beta-D-glucosaminidase involved in a novel chitinolytic pathway from the hyperthermophilic archaeon Thermococcus kodakaraensis KOD1 J Bacteriol 2003 185 17 5175 5181 10.1128/JB.185.17.5175-5181.2003 12923090 PMC181003

[B34] Ferrer M Golyshina OV Plou FJ Timmis KN Golyshin PN A novel alpha-glucosidase from the acidophilic archaeon Ferroplasma acidiphilum strain Y with high transglycosylation activity and an unusual catalytic nucleophile Biochem J 2005 391 Pt 2 269 276 15954864 10.1042/BJ20050346 PMC1276924

[B35] Cantarel BL Coutinho PM Rancurel C Bernard T Lombard V Henrissat B The Carbohydrate-Active EnZymes database (CAZy): an expert resource for Glycogenomics Nucleic Acids Res 2009 37 Database D233 238 10.1093/nar/gkn663 18838391 PMC2686590

[B36] Waterston RH Lindblad-Toh K Birney E Rogers J Abril JF Agarwal P Agarwala R Ainscough R Alexandersson M An P Initial sequencing and comparative analysis of the mouse genome Nature 2002 420 6915 520 562 10.1038/nature01262 12466850

[B37] Li LL McCorkle SR Monchy S Taghavi S Lelie D van der Bioprospecting metagenomes: glycosyl hydrolases for converting biomass Biotechnol Biofuels 2009 2 10 10.1186/1754-6834-2-10 19450243 PMC2694162

[B38] Fukuda M Watanabe S Kaneko J Itoh Y Kamio Y The membrane lipoprotein LppX of Paenibacillus sp. strain W-61 serves as a molecular chaperone for xylanase of glycoside hydrolase family 11 during secretion across the cytoplasmic membrane J Bacteriol 2009 191 5 1641 1649 10.1128/JB.01285-08 19103919 PMC2648208

[B39] Ito Y Tomita T Roy N Nakano A Sugawara-Tomita N Watanabe S Okai N Abe N Kamio Y Cloning, expression, and cell surface localization of Paenibacillus sp. strain W-61 xylanase 5, a multidomain xylanase Appl Environ Microbiol 2003 69 12 6969 6978 10.1128/AEM.69.12.6969-6978.2003 14660338 PMC310030

[B40] Cann IK Kocherginskaya S King MR White BA Mackie RI Molecular cloning, sequencing, and expression of a novel multidomain mannanase gene from Thermoanaerobacterium polysaccharolyticum J Bacteriol 1999 181 5 1643 1651 10049399 10.1128/jb.181.5.1643-1651.1999 PMC93557

[B41] Liu SY Gherardini FC Matuschek M Bahl H Wiegel J Cloning, sequencing, and expression of the gene encoding a large S-layer-associated endoxylanase from Thermoanaerobacterium sp. strain JW/SL-YS 485 in Escherichia coli J Bacteriol 1996 178 6 1539 1547 8626279 10.1128/jb.178.6.1539-1547.1996 PMC177836

[B42] Doi RH Kosugi A Murashima K Tamaru Y Han SO Cellulosomes from mesophilic bacteria J Bacteriol 2003 185 20 5907 5914 10.1128/JB.185.20.5907-5914.2003 14526000 PMC225047

[B43] Freigang J Proba K Leder L Diederichs K Sonderegger P Welte W The crystal structure of the ligand binding module of axonin-1/TAG-1 suggests a zipper mechanism for neural cell adhesion Cell 2000 101 4 425 433 10.1016/S0092-8674(00)80852-1 10830169

[B44] Berman HM Westbrook J Feng Z Gilliland G Bhat TN Weissig H Shindyalov IN Bourne PE The Protein Data Bank Nucleic Acids Res 2000 28 1 235 242 10.1093/nar/28.1.235 10592235 PMC102472

[B45] Zhang Y Skolnick J TM-align: a protein structure alignment algorithm based on the TM-score Nucleic Acids Res 2005 33 7 2302 2309 10.1093/nar/gki524 15849316 PMC1084323

[B46] Zhang Y Skolnick J Scoring function for automated assessment of protein structure template quality Proteins 2004 57 4 702 710 10.1002/prot.20264 15476259

[B47] Doi RH Kosugi A Cellulosomes: plant-cell-wall-degrading enzyme complexes Nat Rev Microbiol 2004 2 7 541 551 10.1038/nrmicro925 15197390

